# Anti-Müllerian hormone and pregnancy after autologous hematopoietic stem cell transplantation for multiple sclerosis

**DOI:** 10.1371/journal.pone.0284288

**Published:** 2023-04-12

**Authors:** Lida Zafeiri, Torbjörn Åkerfeldt, Andreas Tolf, Kristina Carlson, Alkistis Skalkidou, Joachim Burman

**Affiliations:** 1 Department of Medical Sciences, Uppsala University, Uppsala, Sweden; 2 Department of Women’s and Children’s Health, Uppsala University, Uppsala, Sweden; San Raffaele Institute: IRCCS Ospedale San Raffaele, ITALY

## Abstract

Autologous hematopoietic stem cell transplantation (AHSCT) has been approved for multiple sclerosis (MS) in many European countries. A large proportion of patients are women of child-bearing age. For them, AHSCT may have negative consequences for reproductive health, since the ovaries are particularly susceptible to alkylating agents. Anti-Müllerian hormone (AMH) reflects the ovarian reserve and has been suggested as a potential biomarker of fertility in women. The aim of this study was to investigate AMH levels in relation to age and reproductive potential in MS patients treated with AHSCT. The study cohort comprised 38 female patients, aged 20–44 years, who underwent AHSCT for MS using a cyclophosphamide (200 mg/kg)/rabbit—anti-thymocyte globulin (6 mg/kg) conditioning regimen between 2013–2020. Clinal follow-up visits were made 3 months after AHSCT and then yearly. AMH was analysed in blood samples. The median age at transplantation was 28 years (interquartile range, IQR 25–33). The median AMH concentration was 23 pmol/l at baseline (IQR 6.0–30), 0.5 pmol/l at 3 months (IQR 0–1.5) and 1.1 pmol/l at 2 years (IQR 0–2.9). A multiple linear regression model was used to determine if age and/or AHSCT influenced AMH values; both significantly did (age, -0.21 per year, p = 0.018; AHSCT -19, p <0.0001). Seven women became pregnant, six spontaneously and one both spontaneously and with IVF. One patient underwent an abortion, all other pregnancies led to live births. Six of the women became pregnant despite low or very low post-AHSCT serum concentrations of AMH, suggesting that low serum AMH concentrations do not necessarily reflect impaired fertility in patients treated with high-dose cyclophosphamide.

## Introduction

Multiple sclerosis (MS) is an inflammatory disease of the central nervous system, affecting an estimated 2.8 million individuals world-wide [1]. An increasing number of patients are undergoing autologous hematopoietic stem cell transplantation (AHSCT) for MS and the European Society for Blood and Marrow Transplantation (EBMT) registry now includes more than 1800 patients with MS who received AHSCT [2]. The evidence base convincingly demonstrates robust clinical efficacy along with acceptable safety. With appropriate patient selection, 66–93% maintain ‘no evidence of disease activity’ over several years [2]. Patients with a relapsing-remitting disease course, younger age, shorter duration of the disease, inflammatory activity on MR scans, low level of fixed disability and absence of other comorbidities are more likely to benefit from the procedure [2–4]. A large proportion of patients treated with AHCST for MS are women of child-bearing age. For them, AHSCT may have negative consequences for reproductive health, as the ovaries are particularly susceptible to alkylating agents such as cyclophosphamide or melphalan, commonly used in conditioning regimens of AHSCT for MS [4, 5].

Anti-Müllerian hormone (AMH) is thought to reflect the ovarian reserve and several authors have proposed it as a potential biomarker of fertility in women [6, 7]. It is mainly expressed in ovarian follicles and is secreted within the follicular fluid and then into the circulation [6, 7]. It is a regulator of folliculogenesis, determining the number of growing follicles and their selection for ovulation by inhibiting the early stages of follicular development and making them less sensitive to the effects of follicle stimulating hormone [6, 7]. AMH expression increases up to 25 years of age, when it reaches its maximum and then decreases until menopause, when it no longer can be detected in blood [6, 8]. It can be measured at any timepoint of the menstrual cycle as intra- and inter-cycle variability is considered to be low [6, 7]. The European Society of Human Reproduction and Embryology proposes that AMH levels below 0.5–1.1 ng/ml (3.6–7.9 pmol/l) indicate a poor ovarian reserve [9]. In women of reproductive age (between 25 and 40 years), the literature suggests AMH concentrations of 1.0 to 3.0 ng/ml (7.1–21.4 pmol/l) as “normal”, 0.7 to 0.9 ng/ml (5.0–6.4 pmol/l) as “low normal”, and 0.3 to 0.6 ng/ml (2.1–4.3 pmol/l) as “low” and less than 0.3 ng/ml (2.1 pmol/l) as “very low” [10].

The aim of the present study was to investigate AMH levels in relation to age and reproductive potential in MS patients treated with AHSCT.

## Materials and methods

We performed a single-center study at the outpatient clinic of the Department of Neurology at Uppsala University Hospital, Sweden. The study was approved by the Swedish Ethical Review Authority (Dnr 2012/080/1) and was performed in concordance with the Declaration of Helsinki (1964). Written consent was obtained from all patients. Statistical analyses were made with GraphPad Prism 9.

The study cohort comprised 38 female patients, aged 20–44 years, who underwent AHSCT for MS using a Cy (200 mg/kg)/rabbit—ATG (6 mg/kg) conditioning regimen between 2013–2020. None of the patients had a prior history of treatment with chemotherapy. Clinical follow-up visits were made 3 months after AHSCT and then yearly. AMH was analysed in blood samples with an Elecsys AMH Plus automated one-step sandwich chemiluminescence immunoassay on a Cobas Pro e801 module (Roche Diagnostics).

## Results

**Fig 1:**
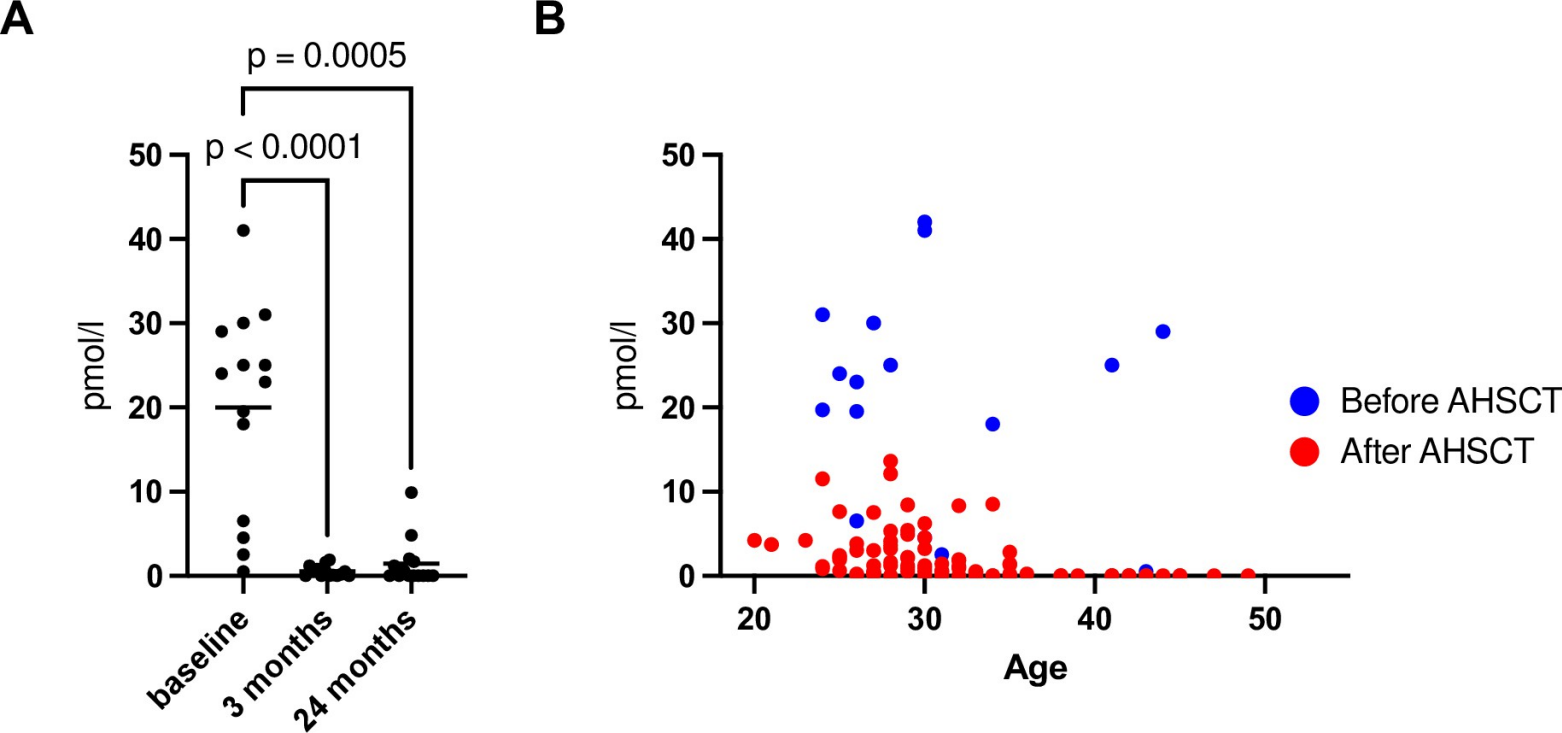
Anti-Müllerian hormone concentrations in serum from patients treated with autologous hematopoietic stem cell transplantation for multiple sclerosis. **(A)** AMH was measured at baseline, 3 months after AHSCT and 24 months after AHSCT. A large decrease in AMH concentrations was seen already at 3 months and present throughout the follow-up period. **(B)** AMH in relation to age before and after treatment with AHSCT. Both age and AHSCT impacted the serum concentrations of AMH and no recovery of AMH was seen in patients older than 35 years of age.

The median age at transplantation was 28 years (interquartile range, IQR 25–33). The median EDSS before transplantation was 3.5 (IQR 2–4). The median AMH concentration was 23 pmol/l at baseline (IQR 6.0–30), 0.5 pmol/l at 3 months (IQR 0–1.5), 0.9 pmol/l at 1 year (IQR 0.25–2.9), 1.1 pmol/ml at 2 years (IQR 0–2.9), 1.2pmol/l at 3 years (IQR 0–4.7), 1.1pmol/l at 4 years (IQR 0–3.7), 1.6 pmol/l at 5 years (IQR 0.15–5.6). Pairwise comparisons were made for the AMH values at baseline, 3 months, and 2 years using the Friedman test for the 14 patients with a complete set of data (Fig 1A). A multiple linear regression model was used to determine if age and/or AHSCT influenced AMH values (Fig 1B); both significantly affected AMH (age, -0.21 per year, p = 0.018; AHSCT -19, p <0.0001).

Seven patients became pregnant, six spontaneously and one both spontaneously and with IVF. The median age at conception was 29 years (IQR 27.5–30) with conception in the range 12–61 months after AHSCT. Pre-AHSCT AMH concentrations were normal (range 20–31 pmol/l). Despite very low post AHSCT AMH concentrations (range 0–1.7 pmol/l) in four and low (range 2.0–4.5 pmol/l) in two of these patients, spontaneous pregnancies were achieved in these six women. Of the rest, one had normal concentrations (8.4 pmol/l). One patient underwent an abortion, all other pregnancies led to live births.

## Discussion

To our knowledge, this is the first study investigating AMH levels and the reproductive potential in MS patients treated with AHSCT using the Cy/ATG conditioning regimen. A key finding of this study is that serum concentrations of AMH were lowered up to five years after AHSCT. However, despite gonadotoxicity, expressed as low or very low post-AHSCT serum concentrations of AMH, six of these women became pregnant spontaneously and delivered healthy offspring.

In the literature, there are few studies investigating fertility and pregnancy outcomes after AHSCT for multiple sclerosis. In a recent study of a mixed population of women with MS treated with bis-chloroethylnitrosourea + etoposide + cytosine-arabinoside + melphalan (BEAM)/anti-thymocyte globulin (ATG) or cyclophosphamide (Cy)/ATG, 30/43 women recovered menstruation after AHSCT. AMH was measured in 13/43 patients after AHSCT and was low in all patients. Four of the women with normal menstrual function tried to conceive, three of them achieving spontaneous pregnancies [11]. In another study published in the same year, thirty patients of childbearing age with multiple sclerosis underwent AHSCT with the BEAM/ATG conditioning regimen. Four pregnancies in three women occurred following AHSCT. One of these women had amenorrhea, one oligomenorrhea and one had normal menses. Two pregnancies were carried to term. No maternal or neonatal complications were reported in either case [12]. A retrospective study of the EBMT working party included 324 female patients that were treated with AHSCT for autoimmune diseases, including MS. Twenty-two pregnancies (20 spontaneous) were reported among 15 patients, resulting in live birth in 15/22 pregnancies. No developmental or congenital abnormalities were observed in the offspring [5]. These results show that despite AHSCT gonadotoxicity, some patients can become pregnant.

The Cy/ATG regimen in MS has been increasingly used since the EBMT 2012 guidelines have been published, while the BEAM/ATG has also been maintained [2]. It has been suggested that the Cy/ATG may impair fertility less in comparison to the BEAM/ATG conditioning regimen [12]. Massarotti et al. did not observe a higher risk of amenorrhea among patients treated with BEAM/ATG conditioning regimen, but this study was not sufficiently powered to detect a difference [11]. This hypothesis needs to be further studied in order to answer this question.

Some limitations of this study are the small sample size, the lack of AMH values at some follow-up visits for some of the included patients and the lack of information about the menstrual function at the point of conception. Therefore, our findings require further validation in larger cohorts.

## Conclusion

Our study suggests that low serum AMH concentrations do not necessarily reflect impaired fertility in patients treated with high-dose cyclophosphamide. This finding should be taken into account when addressing fertility issues in this population.

## Supporting information

S1 File Multiple linear regression of AMH, age, transplantation. (CSV)

## References

[1] Prevalence and severity of MS across the world–can new research explain the patterns?—MS International Federation. [cited 2 Nov 2022]. Available: https://www.msif.org/news/2022/09/01/prevalence-and-severity-of-ms-across-the-world-can-new-research-explain-the-patterns/

[2] Sharrack B, Saccardi R, Alexander T, Badoglio M, Burman J, Farge D, et al. Autologous haematopoietic stem cell transplantation and other cellular therapy in multiple sclerosis and immune-mediated neurological diseases: updated guidelines and recommendations from the EBMT Autoimmune Diseases Working Party (ADWP) and the Joint Acc. Bone Marrow Transplant. 2020;55: 283–306. doi: 10.1038/s41409-019-0684-0 31558790 PMC6995781

[3] Muraro PA, Martin R, Mancardi GL, Nicholas R, Sormani MP, Saccardi R. Autologous haematopoietic stem cell transplantation for treatment of multiple sclerosis. Nat Rev Neurol. 2017;13: 391–405. doi: 10.1038/nrneurol.2017.81 28621766

[4] Das J, Sharrack B, Snowden JA. Correction to: Autologous Haematopoietic Stem Cell Transplantation in Multiple Sclerosis: a Review of Current Literature and Future Directions for Transplant Haematologists and Oncologists (Current Hematologic Malignancy Reports), (2019), 14, 2, (127–135),. Curr Hematol Malig Rep. 2019;14: 136. doi: 10.1007/s11899-019-00506-y 31030389 PMC6828055

[5] Snarski E, Snowden JA, Oliveira MC, Simoes B, Badoglio M, Carlson K, et al. Onset and outcome of pregnancy after autologous haematopoietic SCT (AHSCT) for autoimmune diseases: A retrospective study of the EBMT autoimmune diseases working party (ADWP). Bone Marrow Transplant. 2015;50: 216–220. doi: 10.1038/bmt.2014.248 25387098

[6] Bedenk J, Vrtačnik-Bokal E, Virant-Klun I. The role of anti-Müllerian hormone (AMH) in ovarian disease and infertility. J Assist Reprod Genet. 2020;37: 89–100. doi: 10.1007/s10815-019-01622-7 31755000 PMC7000586

[7] La Marca A, Sighinolfi G, Radi D, Argento C, Baraldi E, Artenisio AC, et al. Anti-Müllerian hormone (AMH) as a predictive marker in assisted reproductive technology (ART). Hum Reprod Update. 2009;16: 113–130. doi: 10.1093/humupd/dmp036 19793843

[8] Kruszynska A, Slowinska-Srzednicka J. Anti-Müllerian hormone (AMH) as a good predictor of time of menopause. Prz Menopauzalny. 2017;16: 47–50. doi: 10.5114/pm.2017.68591 28721129 PMC5509971

[9] Ferraretti AP, La Marca A, Fauser BCJM, Tarlatzis B, Nargund G, Gianaroli L. ESHRE consensus on the definition of “poor response” to ovarian stimulation for in vitro fertilization: the Bologna criteria. Hum Reprod. 2011;26: 1616–1624. doi: 10.1093/humrep/der092 21505041

[10] Jamil Z, Fatima SS, Ahmed K, Malik R. Anti-Mullerian Hormone: Above and beyond Conventional Ovarian Reserve Markers. Dis Markers. 2016;2016. doi: 10.1155/2016/5246217 26977116 PMC4764725

[11] Massarotti C, Sbragia E, Boffa G, Vercelli C, Zimatore GB, Cottone S, et al. Menstrual cycle resumption and female fertility after autologous hematopoietic stem cell transplantation for multiple sclerosis. Mult Scler. 2021;27: 2103–2107. doi: 10.1177/13524585211000616 33709839

[12] Chatterton S, Withers B, Sutton IJ, Milliken ST, Ma DD, Moore JJ, et al. Pregnancy post autologous stem cell transplant with BEAM conditioning for multiple sclerosis. Mult Scler J. 2021; 135245852110056. doi: 10.1177/13524585211005660 33870788

